# Elevated Anti-SARS-CoV-2 Antibodies and IL-6, IL-8, MIP-1β, Early Predictors of Severe COVID-19

**DOI:** 10.3390/microorganisms9112259

**Published:** 2021-10-29

**Authors:** Helena Codina, Irene Vieitez, Alicia Gutierrez-Valencia, Vasso Skouridou, Cristina Martínez, Lucía Patiño, Mariluz Botero-Gallego, María Trujillo-Rodríguez, Ana Serna-Gallego, Esperanza Muñoz-Muela, María M. Bobillo, Alexandre Pérez, Jorge Julio Cabrera-Alvar, Manuel Crespo, Ciara K. O’Sullivan, Ezequiel Ruiz-Mateos, Eva Poveda

**Affiliations:** 1 Group of Virology and Pathogenesis, Galicia Sur Health Research Institute (IIS Galicia Sur), SERGAS-UVigo, 36213 Vigo, Spain; helena.codina@iisgaliciasur.es (H.C.); lucia.patino@iisgaliciasur.es (L.P.); maria.marcos@iisgaliciasur.es (M.M.B.); alexandre.perez@iisgaliciasur.es (A.P.); 2 Rare Diseases & Pediatric Medicine Research Group, Galicia Sur Health Research Institute (IIS Galicia Sur), SERGAS-Uvigo, 36213 Vigo, Spain; irene.vieitez@iisgaliciasur.es; 3 Clinic Unit of Infectious Diseases, Microbiology and Preventive Medicine, Institute of Biomedicine of Seville, IBiS, Virgen del Rocío University Hospital/CSIC/University of Seville, 41013 Seville, Spain; Alicia.gutierrez.valencia@gamil.com (A.G.-V.); maria_tr_5@hotmail.com (M.T.-R.); anasernagallego@gmail.com (A.S.-G.); esperanzamunnozm@gmail.com (E.M.-M.); ezequiel.ruizmateos@gmail.com (E.R.-M.); 4 INTERFIBIO Consolidated Research Group, Departament d’ Enginyeria Quimica, Universitat Rovira i Virgili, 43003 Tarragona, Spain; Vasso.Skouridou@urv.cat (V.S.); Mariluz.Botero@urv.cat (M.B.-G.); ciara.osullivan@urv.cat (C.K.O.); 5 Methodology and Statistics Unit, Galicia Sur Health Research Institute (IIS Galicia Sur)-Complexo Hospitalario Universitario de Vigo, SERGAS-UVigo, 36213 Vigo, Spain; cristina.martinez@iisgaliciasur.es; 6 Infectious Diseases Unit, Department of Internal Medicine, Complexo Hospitalario Universitario de Vigo, IIS Galicia Sur, SERGAS-UVigo, 36213 Vigo, Spain; manuel.crespo.casal@sergas.es; 7 Microbiology Service, Galicia Sur Health Research Institute (IIS Galicia Sur), SERGAS-Uvigo, 36213 Vigo, Spain; Jorge.julio.cabrera.alvargonzalez@sergas.es; 8 Institució Catalana de Recerca i Estudis Avançats, 08010 Barcelona, Spain

**Keywords:** SARS-CoV-2, severe COVID-19, anti-SARS-CoV-2 antibodies, cytokines, viral load

## Abstract

Viral and host immune kinetics during acute COVID-19 and after remission of acute symptoms need better characterization. SARS-CoV-2 RNA, anti-SARS-CoV-2 IgA, IgM, and IgG antibodies, and proinflammatory cytokines were measured in sequential samples from hospitalized COVID-19 patients during acute infection and six months following diagnosis. Twenty four laboratory confirmed COVID-19 patients with mild/moderate and severe COVID-19 were included. Most were males (83%) with a median age of 61 years. Twenty one percent were admitted to the intensive care unit (ICU) and eight of them (33.3%) met the criteria for severe COVID-19 disease. A delay in SARS-CoV-2 levels’ decline during the first six days of follow up, and viral load persistence until month 3 were related to severe COVID-19, but not viral load levels at the diagnosis. Higher levels of anti-SARS-CoV-2 IgA, IgM, IgG and the cytokines IL-6, IL-8 and MIP-1β at the diagnosis time were related to the severe COVID-19 outcome. Higher levels of MIP-1β, IL-1β, MIP-1α and IFN-γ were observed at month 1 and 3 during mild/moderate disease, compared to severe COVID-19. IgG persisted at low levels after six months of diagnosis. In conclusion, higher concentrations of IgA, IgM, and IgG, and IL-6, IL-8 and MIP-1β are identified as early predictors of COVID-19 severity, whereas no significant association is found between baseline SARS-COV-2 viral load and COVID-19 severity.

## 1. Introduction

Coronavirus disease 2019 (COVID-19), caused by severe acute respiratory syndrome coronavirus 2 (SARS-CoV-2), rapidly spread worldwide, becoming a global public health emergency. COVID-19 can be asymptomatic or mild in most cases, but it can rapidly progress to a severe lung inflammation leading to acute respiratory distress syndrome (ARDS), especially in older adults (>80 years) and/or those with comorbidities (i.e., serious heart conditions, chronic pulmonary disease, diabetes mellitus, or hypertension among others) [1,2,3]. The disease outcome mainly depends on the characteristics of the viral replication and host immune responses, which can end up resolving the infection efficiently or creating an exacerbated inflammation associated with severe lung damage pathology, organ failure and poor outcomes. Regarding the host immune responses, SARS-CoV-2 antibody levels follow a general pattern: IgM and IgA are detectable at day 2–5 post symptom onset and levels decrease at week 3, whereas IgG responds later than IgA and IgM, being detectable around 10 days after the onset of symptoms and the maximum levels are reached at 30–35 days [4,5,6]. Although the dynamics of IgM, IgA and IgG during the first weeks of infection have been broadly described, there are few studies reporting results beyond 40 days after symptom onset [7,8]. Finally, another important factor related to the severity of COVID-19 is the so called cytokine storm. The severe deterioration of some patients is closely related to the excessive and prolonged cytokine and chemokine responses induced by the SARS-CoV-2 virus [9]. Higher serum levels of proinflammatory cytokines have been observed in many patients with severe COVID-19, compared with individuals with mild disease [10,11]. The dynamics of proinflammatory cytokines during the first weeks of COVID-19 infection has also been reported, but long term analyses are still lacking.

Therefore, the comprehensive long term kinetics of SARS-CoV-2 RNA levels, anti-SARS-CoV-2 antibodies (i.e., IgA, IgM, and IgG) and specific proinflammatory cytokines during and after COVID-19 are not fully characterized, and these are of great interest for the identification of early predictive biomarkers of severe disease and to understand the clinical outcomes of patients after the remission of acute symptoms.

This study comprises an evaluation of clinical, virological and immunological responses in a well characterized cohort of hospitalized COVID-19 patients with mild/moderate and severe disease. We performed a close follow up at different time points, from the time of diagnosis and up to 6 months after the confirmation of the SARS-CoV-2 infection. Moreover, we were able to identify viral and host immune biomarkers associated with COVID-19 severity. A delay in SARS-CoV-2-RNA clearance in the upper respiratory tract during the first days of the disease, higher concentrations of anti-SARS-CoV-2 IgA, IgM, and IgG and of specific cytokines (i.e., IL-6, IL-8 and MIP-1β) at baseline were associated with COVID-19 severity. Overall, after 6 months, anti-SARS-CoV-2 IgG persisted, although at low levels.

## 2. Materials and Methods

### 2.1. Patients, Sample Collection and Clinical Data

The study population was selected from the COVID-19 Cohort of the Galicia Sur Health Research Institute (COHVID-GS) (https://www.iisgaliciasur.es/apoyo-a-la-investigacion/cohorte-covid19/, accessed on 15 April 2020). This cohort includes laboratory confirmed SARS-CoV-2 patients in clinical follow up at the Vigo Healthcare Area with epidemiological/clinical data and with a repository of biological samples (i.e., nasopharyngeal swabs, serum, plasma and peripheral blood mononuclear cells-PBMCs) stored at the Galicia Sur Health Research Institute Biobank. The epidemiological/clinical information was collected in a case report form (CRF) specifically predesigned for the COHVID-GS. The cohort also includes a control group of uninfected individuals (anti-IgA, IgM and IgG SARS-CoV-2 negative). The study was approved by the Galician Clinical Research Ethics Committee (CEIm-g, ref: 2020/196, signed on 10 April 2020) and all patients signed the informed consent. All the techniques were performed in BLS-2 conditions, according to the biosafety guidelines for handling and processing specimens associated with COVID-19 (https://www.cdc.gov/coronavirus/2019-ncov/lab/lab-biosafety-guidelines.html, accessed on 25 January 2021).

The selection criteria for this study were SARS-CoV-2 adult hospitalized patients with a very close clinical follow up with nasopharyngeal swabs, serum and plasma samples, with consecutive samples available at least at baseline, day 3 or 6, and month 1 or 3 from their inclusion in the COHVID-GS. Moreover, only those patients included in the COHVID-GS between day 1 and 5, following the confirmation of the SARS-CoV-2 infection by RT-qPCR, were considered. Serial paired nasopharyngeal swabs, and serum and plasma samples were collected from each patient at baseline, day 3 or 6, month 1 or 3 and at month 6. We also included 30 serum samples from healthy and noninfected individuals as controls for the immunoassay experiments. Epidemiological and clinical data were recorded for the study population.

Following WHO guidance, severe COVID-19 was defined as the need for invasive mechanical ventilation, the development of acute respiratory distress syndrome (PO_2_/FiO_2_ < 300 mmHg or Saturation O_2_/FiO_2_ < 330) or admission to an intensive care unit (ICU) [12]. Patients who did not meet severe criteria were considered mild/moderate COVID-19.

### 2.2. SARS-CoV-2 RNA Extraction

Viral RNA was extracted from 140 µL of nasopharyngeal swab samples using the QIAmp viral RNA mini kit (QIAGEN, Hilden, Germany) and the automatized QIAcube system (QIAGEN, Hilden, Germany), and was eluted in 50 µL of buffer following the manufacturer’s instructions. The positive (EDX SARS-CoV-2 Standard, Exact Diagnostics, Forth Worth, TX, USA) and negative (EDX SARS-CoV-2 Negative, Exact Diagnostics, Forth Worth, TX, USA) controls were also extracted using the same procedure.

### 2.3. Droplet Digital PCR Analysis

SARS-CoV-2 viral load was quantified by reverse transcriptase droplet digital PCR (RT-ddPCR), including the one-step reverse transcription (One-Step RT-ddPCR Advanced Kit for Probes, Bio-Rad Laboratories, Hercules, CA, USA) and the triplex probe assay for PCR amplification (2019-nCoV CDC ddPCR Triplex Probe Assay, Bio-Rad Laboratories, Hercules, CA, USA). The assay contains primers and probes targeting two regions of the SARS-CoV-2 nucleocapsid gene (N1 and N2) and the human Rnase P gene (RPP30). The reaction mixture was performed with 5.5 µL of SARS-CoV-2 RNA sample and following the manufacturer’s instructions. All the samples were tested in duplicate. Data analysis was performed using the QuantaSoft Analysis Pro Software (v. 1.0.596, Bio-Rad Laboratories, Hercules, CA, USA), which showed the results as copies per microliter of 1x ddPCR reaction. All viral load values were recalculated to copies per milliliter of swab, and the sensitivity threshold was 100 copies/mL. To assess the accuracy of the absolute viral RNA quantification, two fold serial dilutions of the positive control were analyzed for lineal regression analysis. Finally, to establish a reliable comparison between the viral load of different samples, the absolute quantification of SARS-CoV-2 RNA was normalized with RPP30 and expressed as copies/10^4^ cells (Figure S1).

### 2.4. Anti-SARS-CoV-2 IgA, IgG, and IgM Quantification

The wells of 96-well immunoassay plates (MaxiSorp-Nunc, Thermo Fisher Scientific Inc, Waltham, MA, USA) were coated overnight at 4 °C with 50 μL of 5 μg/mL of SARS-CoV-2 nucleoprotein (NP) (ref. MBS596190 – MyBiouSource Inc, San Diego, CA, USA) in 50 mM carbonate buffer pH 9.4. The wells were washed three times with 200 μL of PBS containing 0.05% (*v*/*v*) Tween-20 (PBST) and then blocked with 200 μL of 5% (*w*/*v*) skim milk in PBST for 30 min. After another washing step, 50 μL of serum samples (diluted 1/100 with PBS after heating for 30 min at 56 °C to inactivate residual virus) were added to the wells and incubated for 1 h. The wells were washed again three times with PBST and 50 μL of antihuman IgA-HRP (ref. PA174395—Thermo Fisher Scientific Inc, Waltham, MA, USA), antihuman IgM-HRP (ref. 31415—Thermo Fisher Scientific Inc, Waltham, MA, USA) or antihuman IgG-HRP (ref. A0170—Sigma, San Luis, MO, USA) enzyme conjugates diluted 1/20000 with PBST were added. After a final incubation for 30 min, the wells were washed five times with PBST and 50 μL of TMB Super Sensitive ELISA substrate was added in each well. The reaction was stopped by the addition of 50 μL of 1 M H_2_SO_4_ after 5 min, for IgM and IgA detection, or 7 min, for IgG detection. The absorbance at 450 nm was finally read on a SPECTRAmax 340PC-384 microplate reader. The levels of IgA, IgM and IgG antibodies in each sample were estimated using standard antibody calibration curves. Particularly, serial dilutions of each antibody, standard IgA (ref. 31148—Thermo Fisher Scientific Inc, Waltham, MA, USA), IgM (ref. 31146) or IgG (MP Biomedical—ref. 0855908—Thermo Fisher Scientific Inc, Waltham, MA, USA), were performed with 50 mM carbonate buffer pH 9.4 (dilution factor 1/4) in the range of 3.9 ng/mL–16 μg/mL for IgA and IgG and 1.9 ng/mL–8 μg/mL for IgM, and were used to coat duplicate wells in parallel with the NP coating step for sample analysis. The serum samples were analyzed on NP-coated wells in triplicate, while duplicate control (noncoated) wells were also included to eliminate any signals resulting from nonspecific binding of serum components to the wells. Calibration curves for each antibody type (IgA, IgM or IgG) were constructed by fitting the absorbance values (A450 nm) to a sigmoidal 4-parameter logistical model using GraphPad Prism. The concentration of each antibody was then interpolated from its corresponding calibration curve using the corrected (A–A0) values for each sample, where A was the average absorbance of the NP-coated wells and A0 the average absorbance of the control (noncoated) wells. Prepandemic serum samples (*n* = 30) were used as controls to set the background values of the in house developed ELISA. All incubation steps were performed at room temperature (22–25 °C) unless stated otherwise.

### 2.5. Plasma Cytokines Quantification

Plasma cytokines concentrations were determined using enzyme linked immunosorbent assays. Interleukin-6 (IL-6), interleukin-8 (IL-8), interleukin 1 beta (IL-1β), tumor necrosis factor-alpha (TNF-α), interferon-gamma (IFN-γ), macrophage inflammatory proteins 1 alpha (MIP-1α) and 1 beta (MIP-1β) were analyzed using a multiplex bead based immunoassay (MILLIPLEX^®^ MAP Human High Sensitivity T Cell Magnetic Bead Panel, Merck KgaA, Darmstadt, DE, USA). Interferon gamma induced protein 10 (IP-10) (Human CXCL10 ELISA kit, Abcam, Cambridge, UK) and soluble receptor of interleukin 2 (sCD25) were analyzed using the Human Quantikine Immunoassay (R&D Systems, Minneapolis, MN, USA). All followed the manufacturer’s instructions.

### 2.6. Statistics

The descriptive analyses were reported as frequencies and percentages for categorical variables, and medians and interquartile ranges (IQRs) for continuous variables. To compare antibody levels between COVID-19 patients and a peer control group, and to compare SARS-CoV-2 viral load and cytokines between severe and mild/moderate patients, Mann–Whitney U test was performed. To assess the differences in the viral load over time, and the concentration of cytokines at several time points during the first 6 months after SARS-CoV-2 infection, a Wilcoxon Signed Rank test was performed. Finally, Mann–Whitney U test was also used to compare the baseline antibodies levels and baseline cytokines concentration with the severe clinical outcomes (ICU admission, requirement of invasive mechanical ventilation and development of Acute Respiratory Distress Syndrome). Statistical analyses were performed with SPSS software (v.19, IBM, Endicott, NY, USA) and GraphPad Prism software (v. 8.2.1, GraphPad, San Diego, CA, USA) and a *p* value less than 0.05 was considered statistical significance.

## 3. Results

### 3.1. Demographic and Clinical Characteristics of the Studied Patients

A total of 24 hospitalized laboratory confirmed COVID-19 patients were included in the study. The demographic and most relevant clinical characteristics related to COVID-19 are shown in Table 1. Most, 83% (20/24), were males, with a median age of 61 years old (IQR: 48–75.3). Nearly 50% (12/24) of patients met obesity criteria (BMI ≥ 30) and 42% (10/24) had hypertension. The median hospitalization time was 8 days, and 21% (5/24) were admitted to the ICU. Eight of them (33.3%) met the criteria for severe COVID-19 disease. 

### 3.2. SARS-CoV-2 Viral Load Kinetics

**Figure 1:**
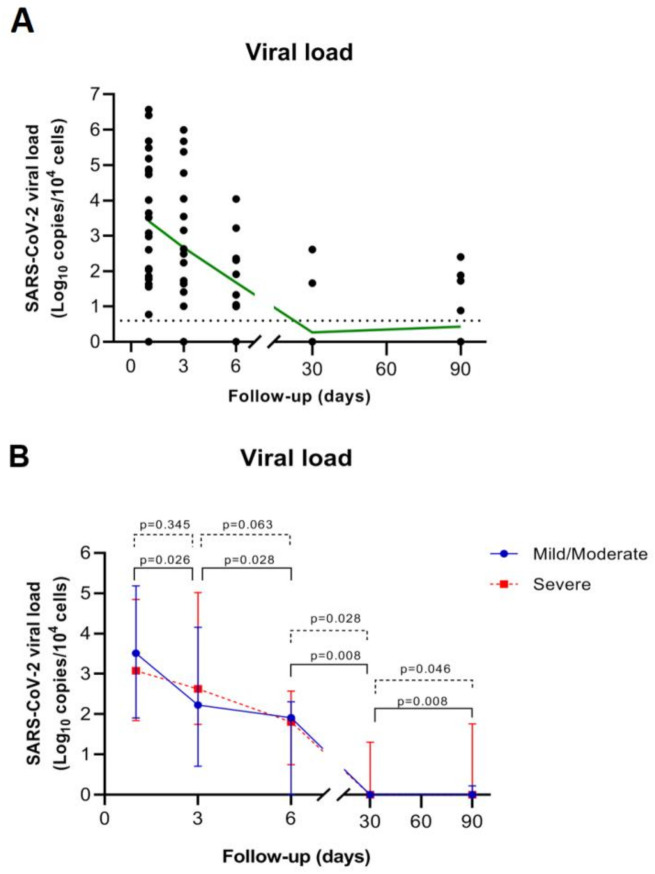
SARS-CoV-2 viral load kinetics. (**A**) SARS-CoV-2 RNA levels among the overall study population during the study period. The black dots represent the aligned individual values obtained from each time point. The green line represents the connected mean values. The dotted line indicates the established experimental threshold (below the lowest positive sample obtained in our study). (**B**) Median SARS-CoV-2 RNA levels in patients with mild/moderate versus severe COVID-19. The solid blue line with circles represents the mild/moderate group and dotted red line with squares represents the severe group. Wilcoxon signed rank test was performed for the decline of viral load between time points; p: *p*-value.

The SARS-CoV-2 viral load quantification was performed from 92 RNA samples extracted from nasopharyngeal swabs in the 24 patients at different time points (24 at baseline, 18 at day 3, 13 at day 6, 5 at day 15, 16 at month 1, and 16 at month 3) (Table 2). At baseline, 23 of 24 patients (96%) were positive for SARS-CoV-2 with a median viral load of 2227.17 copies/10^4^ cells, showing broad variability between patients (IQR: 69.78–7.42 × 10^4^). Only one patient (COV 015) was negative for SARS-CoV-2 RNA quantification, but he had also low amplification of RPP30, indicating a deficient sampling. Overall, SARS-CoV-2 viral load decreased overtime, and most of the patients (80%) were negative one month after the diagnosis (Figure 1A). However, five patients showed persistent viral load at month 1 and 3. All these patients had severe disease (COV 006, COV 009 and COV 013) and/or comorbidities, such as diabetes (COV 016 and COV 013); hypertension or cardiovascular disorders (COV 016, COV 019 and COV 013); chronic lung disease and HIV infection (COV 009) and dyslipidemia (COV 006). Two different profiles in SARS-CoV-2 kinetics were observed during acute infection, based on the COVID-19 clinical outcomes. A delay in the significant decrease in SARS-CoV-2 levels during the first 6 days of follow up was recognized in those patients with severe disease. Thus, while in patients with mild/moderate disease a significant decline in viral load is observed from the diagnosis time, in patients with severe disease, this is only observed after day 6 (Figure 1B). 

### 3.3. Anti-SARS-CoV-2 Antibodies Kinetics

**Figure 2:**
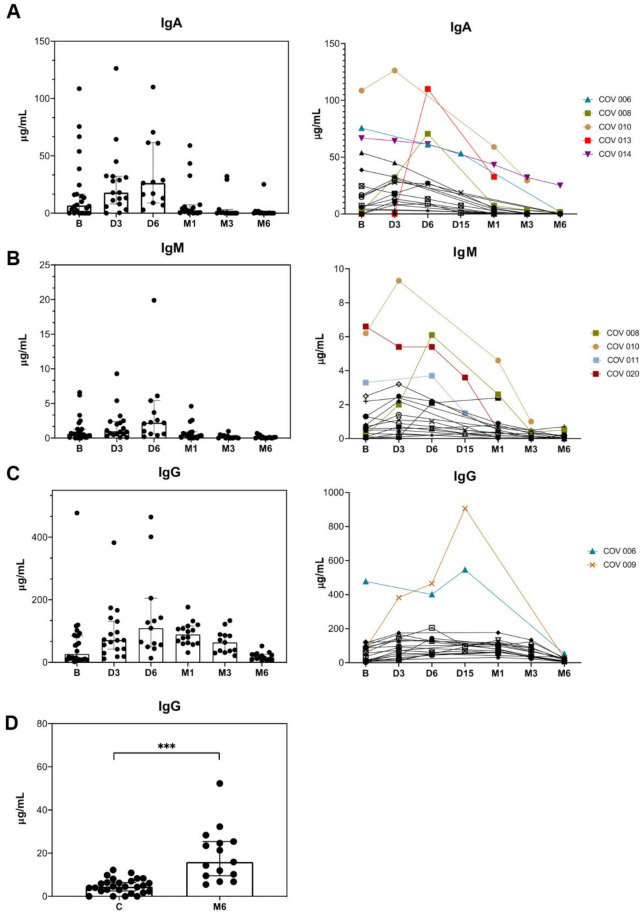
Absolute levels at different time points during the study period and antibodies IgA (**A**), IgM (**B**) and IgG (**C**) kinetics in specific patients. Absolute levels of IgA (**A**), IgM (**B**) and IgG (**C**) among the overall study population (*n* = 24) at baseline (B), day 3 (D3), day 6 (D6), month 1 (M1), month 3 (M3) and month 6 (M6) after SARS-CoV-2 infection and changes of serum IgA (**A**), IgM (**B**) and IgG (**C**) antibodies in specific patients (*n* = 19) at baseline (B), day 3 (D3), day 6 (D6), month 1 (M1), month 3 (M3) and month 6 (M6) after SARS-CoV-2 infection. Patients COV 006, COV 008, COV 009, COV 010, COV 011, COV 013, COV 014 and COV 020 belong to the severe group. (**D**) Comparison of IgG levels between uninfected controls (c) and patients after 6 months of SARS-CoV-2 infection. Mann–Whitney U test. ***, *p*-value < 0.0001.

The anti-SARS-CoV-2 antibodies levels were quantified at different time points, from a total of 109 serum samples (24 at baseline, 18 at day 3, 13 at day 6, 5 at day 15, 16 at month 1, 18 at month 3 and 15 at month 6) (Figure 2). Overall, a high variability in IgA baseline levels was observed among patients that progressively increased, reaching the highest median levels at day 6 of follow-up, and then significantly decreased until month 6 (*p* = 0.028). The highest levels of IgA at baseline and during the follow up were observed in patients with severe COVID-19 (COV 006, COV 008, COV 010, COV 014, COV 013) (Figure 2A). A similar pattern was observed for the kinetics of IgM, with an ongoing increase reaching the maximum levels at day 6 and with a significant decline after that (*p* = 0.028). Similar to IgA, the highest levels of IgM at baseline and during the follow up were observed in patients with severe COVID-19 (COV 008, COV 011, COV 010, COV 020) (Figure 2B). For IgG, there was also a gradual increase until day 6, with a progressive but slow decrease until month 6, reaching very low concentrations (Figure 2C). Of note is that IgG levels at month 6 were significantly higher than in a group of 30 uninfected controls (15.93 vs. 4.50 μg/mL, respectively, *p*-value < 0.001) (Figure 2D). 

The numerical data for IgA, IgM and IgG in serum during the study period are shown as Supplementary Data (Table S1).

### 3.4. Plasma Cytokines Kinetics

**Figure 3:**
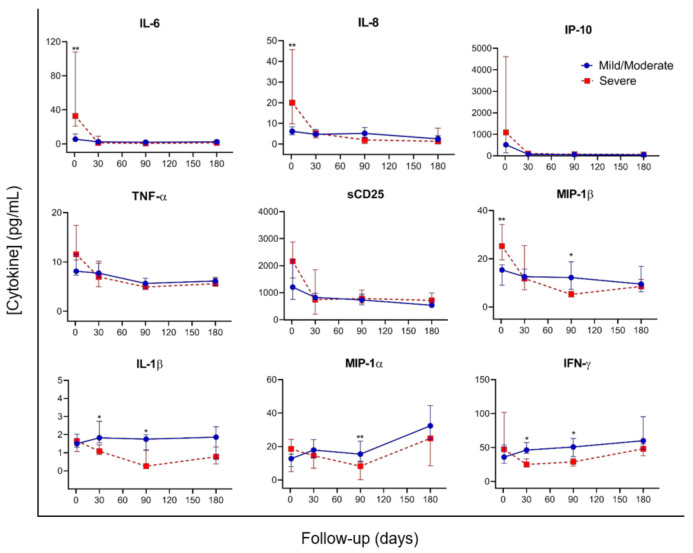
Cytokine kinetics in patients with mild/moderate versus severe COVID-19 during the study period. Cytokine levels are expressed as median ± IQR. The solid blue line with circles represents the mild/moderate group (*n* = 12) and dotted red line with squares represents severe group (*n* = 6). Differences between both groups at each time point were assessed using Mann–Whitney U test. *, *p*-value < 0,05; **, *p*-value < 0.001.

Plasma cytokines levels were performed in a total of 18 patients, 12 with mild/moderate and 6 with severe COVID-19, at different time points (18 at baseline, 15 at month 1, 14 at month 3, and 17 at month 6). Figure 3 shows the dynamic of the median concentrations of the nine cytokines assessed (TNF-α, IL-6, IL-8, IL-1β, MIP-1α, MIP-1β, IFN-γ, sCD25 and IP-10) for each group, based on COVID-19 severity, during the study period. 

At baseline, significant higher levels of IL-6, IL-8, and MIP-1β were observed in patients with severe disease compared with those with mild/moderate COVID-19. Overall, a similar dynamic was observed for some of the proinflammatory markers, IL-6, IL-8, IP-10, TNF-α and sCD25, during the study period. By contrast, the dynamic of MIP-1β, IL-1β, MIP-1α and IFN-γ showed a different profile during the follow up, with significant higher levels at month 1 (IL-1β and IFN-γ) and month 3 (MIP-1β, IL-1β, MIP-1α and IFN-γ) among patients with mild/moderate diseases, compared to those with severe COVID-19. After 6 months of the acute SARS-CoV-2 infection, no differences were found between patients with mild/moderate vs. severe disease. 

The numerical data for plasma cytokines comparison between mild/moderate vs. severe patients are shown as Supplementary Data. 

### 3.5. Clinical, Virological and Host Immune Biomarkers for Severe COVID-19 Outcomes

A univariate analysis was performed to identify potential biomarkers associated with severe clinical outcomes, defined as the requirement of invasive mechanical ventilation, the admission to an intensive care unit (ICU) and the development of acute respiratory distress syndrome (ARDS). Higher levels of IgA, IgM, IgG against SARS-CoV-2 and also the cytokines IL-6, IL-8 and MIP-1β at baseline were observed among patients who developed ARDS compared to those without ARDS. Moreover, higher levels of IL-6 and MIP-1β were related to either the need for invasive mechanical ventilation or ICU admission. Overall, SARS-CoV-2 viral load at baseline was not associated with any of the severe clinical outcome definitions (Table 3).

## 4. Discussion

In this study we report that elevated levels of anti-SARS-CoV-2 antibodies, IgA, IgM, and IgG, as well as specific cytokines, such as IL-6, IL-8, MIP-1β, during SARS-CoV-2 acute infection are associated with severe COVID-19, defined by the development of ARDS or the need for invasive mechanical ventilation or UCI admission. IgG persists after 6 months of diagnosis but at lower concentrations. Whilst the levels of some cytokines (i.e., IL-6, IL-8, IP-10, TNF-α, sCD25) declined one month after the SARS-CoV-2 diagnosis, others (MIP-1β, IL-1β, MIP-1α and IFN-γ) showed higher levels at month 1 and/or 3 in patients with mild/moderate disease compared to those with severe COVID-19. However, 6 months after diagnosis, no differences in cytokines levels between patients with mild/moderate vs. severe disease were observed. Interestingly, although SARS-CoV-2 RNA levels were not associated with the clinical outcome, during the acute infection, a significant delay in SARS-CoV-2 viral load decline was recognized in patients with severe COVID-19 as compared to those with mild/moderate COVID-19 during the first 6 days of follow up. 

Overall, SARS-CoV-2 viral load kinetics follow the pattern described previously, reaching maximum levels during acute infection and decreasing progressively until becoming undetectable in most patients less than one month following diagnosis [13,14,15,16]. However, because of the close follow up of these patients, we were able to identify a significant delay during the first days of viral load decay in patients who met the criteria for severe COVID-19. Nonetheless, in our cohort, SARS-CoV-2 RNA levels were not associated with clinical outcome at any time point. There are some controversial data regarding this issue: while some studies have not reported any association between SARS-CoV-2 viral load during acute infection and COVID-19 severity [17,18], others point out that elevated viral load could be used to identify patients at higher risk for morbidity or severe COVID-19 outcome [19,20]. The controversial results may be explained by the heterogeneity of the studies related to the different characteristics of the study population and/or the methodology used for SARS-CoV-2 quantification and sampling quality (i.e., normalization using copies/cells).

Although 80% of patients showed undetectable viremia one month after diagnosis, in five patients we observed SARS-CoV-2 RNA persistence between month 1 and 3. Interestingly, all of these had severe disease and/or comorbidities (i.e., diabetes, hypertension or cardiovascular disorders, chronic lung disease and HIV infection, dyslipidemia). These observations are in agreement with previous studies that reported an association between SARS-CoV-2 RNA persistence with severe disease [15,21] and comorbidity [19,22,23,24] following remission of the acute symptoms. More recently, Jacobs et al. highlighted the association between SARS-CoV-2 plasma viremia and ICU admission [25], and Tokuyama et al. described SARS-CoV-2 persistence in intestinal enterocytes up to 7 months after symptoms resolutions [26]. Additional studies are required to better understand the clinical significance of SARS-CoV-2 persistence in different compartments (i.e., plasma, gut, upper respiratory tract) and its potential use to monitor COVID-19 patients during early acute infection and/or following the remission of acute symptoms.

We found that elevated levels of anti-SARS-CoV-2 antibodies during acute infection are related to severe COVID-19. Those patients who developed ARDS showed significantly higher levels of IgA, IgM, and IgG, compared to patients without ARDS. These findings are in agreement with previous studies that found an association between anti-SARS-CoV-2 antibodies levels and the need for intubation or death [27,28]. Overall, we observed that anti-SARS-CoV-2 IgA and IgM antibodies decay rapidly after the acute infection phase (i.e., at month 3, 54% for IgA and 77% for IgM showed levels below the limit of detection) but the decline of IgG was less prominent and persisted until month 6. These levels were still significantly higher compared to the levels of uninfected controls (15.93 vs. 4.50 μg/mL, respectively, *p*-value < 0.001). The durability of the immune responses against SARS-CoV-2 infection is a matter of intense interest and still remains unclear. Regarding the humoral responses, recent studies have also reported that IgG levels persist between 6 and 8 months after onset of symptoms [29,30]. However, plasma neutralization activity seems to decrease a few weeks after the onset of the symptoms [31]. Understanding the dynamics of antibodies against SARS-CoV-2 and the persistence of the neutralizing activity is critical to establish correct prevention and vaccination strategies.

COVID-19 severity has been associated with exacerbated inflammation due to a massive release of proinflammatory components [4,9]. We have identified that elevated levels of IL-6, IL-8, and MIP-1β during SARS-CoV-2 acute infection are associated with severe COVID-19, defined by the development of ARDS or the need of invasive mechanical ventilation or ICU admission. Higher levels of IL-6 have been consistently related to severe COVID-19 disease [32,33] and play a pivotal role in the cytokine storm in response to SARS-CoV-2 infection, promoting organ failure and severe lung pathology [34,35,36]. IL-8 leads to the activation and recruitment of neutrophils to the inflammation sites, and has been implicated in inflammatory pulmonary diseases, such as ARDS, chronic obstructive pulmonary disease, and asthma [37,38]. In addition, IL-8 has also been reported as a biomarker in predicting severe status COVID-19 patients [10]. MIP-1β drives the recruitment of a variety of innate and adaptive immune cells, and high concentrations have been reported in the serum and lungs of patients with certain acute respiratory viral infections [39,40]. However, their role during SARS-CoV-2 infection remains unclear. Some studies have reported a higher production of MIP-1β at the transcriptional level in bronchoalveolar lavage cells isolated from the lungs of severe COVID-19 patients [41,42], whilst other studies did not find an association between MIP-1β and severe disease [34,43]. 

The levels of IL-6, IL-8, and MIP-1β significantly decline at month 1 in patients with severe COVID-19, showing during the subsequent follow up levels similar to those found in patients with mild/moderate disease. Overall, a similar dynamic was observed for IP-10, TNF-α and sCD25, during the study period. By contrast, the dynamic of MIP-1β, IL-1β, MIP-1α and IFN-γ showed a different evolution pattern, with a significant increase at month 1 and/or 3 among patients with mild/moderate disease, compared to those with severe COVID-19. No differences were observed in the levels of these cytokines 6 months after the acute SARS-CoV-2 infection, between patients with mild/moderate vs. severe disease. Therefore, the unbalance in the cytokine levels between severe and mild/moderate COVID-19 patients seems to be restored after 6 months of SARS-CoV-2 infection. A previous study by Lucas et al. [44] also reported no differences in the IL-6, IL-8 and IP-10 levels between mild/moderate and severe outcomes after 20 days of follow up. 

This study presents some limitations. First, the relatively small size of the study population is mainly represented by men as, during the first wave of COVID-19 in our institution, 86% of hospitalized patients were men. However, we were able to accomplish a very close follow up of the clinical outcomes, viral and host immune factors in this population allowing interesting observations. Although we have identified IgG after 6 months of SARS-CoV-2 infection, we did not assess their neutralization activity and, therefore, the risk upon new SARS-CoV-2 reinfections. External validation should be assessed to confirm the predictive value of our findings. 

In conclusion, in a well characterized cohort of hospitalized COVID-19 patients with mild/moderate and severe disease and close clinical follow up during and after 6 months of SARS-CoV-2 infection, we have recognized early host immune predictors of severity. Higher levels of IgA, IgM, and IgG and the specific cytokines IL-6, IL-8, and MIP-1β during acute infection were observed in those patients with a severe COVID-19 outcome. Conversely, higher levels of IL-1β and IFN-γ at month 1, and MIP-1β, IL-1β, MIP-1α and IFN-γ at month 3, were observed among patients with mild/moderate diseases, compared to those with severe COVID-19. IgG against SARS-CoV-2 persisted after 6 months of the diagnosis. 

## Figures and Tables

**Table 1:** Demographic and clinical characteristics of the study population.

Characteristics	*n* = 24
Age (years)	61.0 (48.0–75.3)
Sex (male)	83% (20)
*Comorbidities*	
Obesity, BMI > 30	50% (12)
Hypertension	42% (10)
Chronic lung disease	21% (5)
Dyslipidemia	21% (5)
Cardiovascular disease	13% (3)
Diabetes	13% (3)
Hospitalization time (days)	8.0 (7.0–16.3)
ICU admission	21% (5)
ICU length of stay (days)	12.0 (9.0–18.5)
Exitus	4% (1)
Time from symptom onset to hospital admission (days)	9.0 (5.5–13.0)
Time from hospital admission to cohort admission (days)	2.0 (2.0–3.0)
Time from PCR+ to cohort admission (days)	3.0 (2.0–5.0)
*Symptoms on admission*	
Fever	75% (18)
Dyspnea	71% (17)
Cough	67% (16)
Malaise	67% (16)
Diarrhea	42% (10)
Sputum	38% (9)
ARDS *	30% (7)
Myalgia/Arthralgia	30% (7)
Anosmia	25% (6)
Chest pain	25% (6)
*Median laboratory values on admission*	
Leukocytes (cells/mL)	4940.0 (4545.0–6815.0)
Lymphocytes (cells/mL)	955.0 (755.0–1215.0)
Neutrophils (cells/mL)	3725.0 (2860.0–5437.5)
Platelets (×10^9^/L)	166.5 (132.8–248.0)
Creatinine (mg/dL)	0.885 (0.778–1.023)
LDH (UI/L) **	274.0 (208.0–350.0)
Bilirubin (mg/dL) **	0.660 (0.430–0.900)
Oxygen therapy	58% (14)
Invasive mechanical ventilation	21% (5)
Pulmonary infiltrates	92% (22)
*Treatments during hospitalization*	
Hydroxychloroquine	71% (17)
Antibiotics	46% (11)
Immunosuppressive therapy	46% (11)
Corticosteroids	42% (10)
Lopinavir/Ritonavir	42% (10)
Azithromycin	30% (7)

Data are median (IQR), % (*n*). *Acute Respiratory Distress Syndrome. ** *n* = 23.

**Table 2:** SARS-CoV-2 RNA quantification from nasopharyngeal swab samples.

	SARS-CoV-2 RNA (Copies/10^4^Cel *)					
**Case**	**Baseline**	**Day 3**	**Day 6**	**Day 15**	**Month 1**	**Month 3**
COV 001	1.03 × 10^4^	4.70 × 10^5^	202.55	NA	ND	NA
COV 002	3.73 × 10^6^	3.48 × 10^3^	NA	NA	ND	ND
COV 003	42.52	25.75	NA	NA	ND	ND
COV 004	3.27 × 10^3^	171.77	NA	NA	ND	ND
COV 005	107.84	ND	NA	NA	ND	ND
**COV 006**	71.27	NA	228.31	ND	NA	76
COV 007	116.38	43.68	NA	NA	ND	ND
**COV 008**	5.43 × 10^4^	1.10 × 10^4^	21.24	NA	ND	ND
**COV 009**	7.08 × 10^4^	424.73	9.97	13.67	NA	52.8
**COV 010**	69.28	307.3	NA	NA	ND	ND
**COV 011**	1.19 × 10^3^	NA	1.65 × 10^3^	ND	ND	ND
COV 012	4.75 × 10^5^	NA	1.10 × 10^4^	ND	NA	NA
**COV 013**	1.53 × 10^5^	9.81 × 10^5^	195.8	NA	408.3	NA
**COV 014**	35.74	10.16	ND	NA	ND	ND
COV 015	ND	ND	NA	NA	ND	ND
COV 016	7.54 × 10^4^	5.91 × 10^4^	NA	NA	45.43	7.68
COV 017	59.94	NA	ND	NA	ND	ND
COV 018	5.89	ND	NA	NA	ND	ND
COV 019	6.88 × 10^4^	1.42 × 10^3^	NA	NA	NA	251.4
**COV 020**	946.4	53.6	81.3	25.2	ND	NA
COV 021	400	169.51	ND	NA	NA	NA
COV 022	3.09 × 10^5^	NA	202.8	NA	NA	NA
COV 023	2.56 × 10^6^	2.37 × 10^5^	NA	NA	NA	NA
COV 024	4.42 × 10^3^	NA	11.1	NA	NA	NA

* copies/10^4^cel: number of viral RNA copies per 10^4^ cells. ND: not detected. NA: sample not collected. Patients from the COVID-19 severe group are highlighted in bold.

**Table 3:** Biomarkers of severe COVID-19.

	ARDS	No ARDS	*p*-Value	Invasive Mechanical Ventilation/ ICU Admission	No Invasive MechanicalVentilation/ ICU Admission	*p*-Value
SARS-CoV-2 load (Log_10_ copies/10^4^ cells)	2.97 (1.78–4.73)	3.64 (1.92–5.36)	0.357	3.07 (2.70–5.01)	3.51 (1.71–4.88)	0.804
Serum antibodies ^a^ (µg/mL)						
IgA	24.51 (7.25–75.62)	4.42 (0.0–14.32)	**0.013**	6.18 (0–16.63)	7.25 (1.22–50.07)	0.783
IgG	84.94 (57.32–102.03)	14.47 (8.38–57.71)	**0.028**	0.56 (0.10–1.30)	0.59 (0.17–4.96)	0.446
IgM	0.66 (0.59–6.25)	0.43 (0.09–1.06)	**0.047**	17.42 (8.96–86.86)	57.32 (6.47–281.21)	0.836
Plasma cytokines ^b^ (pg/mL)						
IL-6	32.72 (20.78–107.92)	5.44 (2.93–11.63)	**0.002**	38.51 (32.72–172.42)	5.80 (3.72–16.57)	**0.017**
IL-8	19.99 (9.83–45.67)	6.43 (4.64–9.51)	**0.007**	21.56 (15.78–51.42)	7.69 (5.02–10.88)	0.056
IP-10	1083.80 (439.15–4613.25)	515.40 (141.3–633.13)	0.102	1606.00 (1076.40–3347.00)	534.70 (177.65–614.15)	0.100
TNF-α	11.55 (10.40–17.45)	8.12 (7.32–11.56)	0.083	10.96 (10.60–11.92)	10.81 (7.55–11.73)	0.574
sCD25	2160.00 (1541.25–2877.0)	1221.50 (775.33–2806.00)	0.250	1567.00 (1515.00–2247.50)	1465.00 (960.95–2534.50)	0.645
MIP-1β	25.29 (19.52–34.19)	15.37 (8.99–17.51)	**0.001**	30.82 (24.47–37.55)	16.60 (9.98–19.99)	**0.039**
IL-1β	1.65 (1.07–1.77)	1.50 (1.31–2.02)	0.750	1.62 (1.42–1.73)	1.60 (1.30–1.80)	1000
MIP-1α	18.52 (5.02–24.21)	12.74 (7.95–15.47)	0.682	15.11 (10.87–18.52)	13.36 (8.34–19.62)	1.00
IFN-γ	47.59 (36.60–101.63)	35.91 (26.79–53.89)	0.213	42.13 (40.57–77.18)	37.01 (30.6–53.66)	0.301

Data are median (IQR) Mann-Whitney U test was performed. *p*-values < 0.05 were considered significant and are highlighted in bold. ^a^ Performed in the following study population: ARDS *n* = 7; No ARDS *n* = 17; ICU admission *n* = 5; No ICU admission *n* = 19; ^b^ Performed in the following study population: ARDS *n* = 6; No ARDS *n* = 12; ICU admission *n* = 3; No ICU admission *n* = 154.

## Data Availability

Not applicable.

## Supplementary Materials

The following are available online at https://www.mdpi.com/article/10.3390/microorganisms9112259/s1, Figure S1: Linear regression analysis of the RT-ddPCR SARS-CoV-2 assay; Table S1: IgA, IgM, IgG levels at the different points of the study periods; Table S2: Cytokine levels (severe vs. moderate).
