# Supplementary material for: Elevated Anti-SARS-CoV-2 Antibodies and IL-6, IL-8, MIP-1β, Early Predictors of Severe COVID-19
Source: Microorganisms. 2021 Oct 29;9(11):2259. doi: 10.3390/microorganisms9112259 (PMC8624589; doi:10.3390/microorganisms9112259)
Supplement: Supplementary file 1 [file microorganisms-09-02259-s001.zip › microorganisms-1433903-supplementary.pdf]

microorganisms

MDPI

Article

Elevated Anti-SARS-CoV-2 Antibodies and IL-6, IL-8, MIP-1β, Early Predictors of Severe COVID-19

Helena Codina [1,†,‡], Irene Vieitez [2,†,‡], Alicia Gutierrez-Valencia [3], Vasso Skouridou [4], Cristina Martínez [5], Lucía Patiño [1,‡], Mariluz Botero-Gallego [4], María Trujillo-Rodríguez [3], Ana Serna-Gallego [3], Esperanza Muñoz-Muela [3], María M. Bobillo [1,‡], Alexandre Pérez [1,6,‡], Jorge Julio Cabrera-Alvar [7,‡], Manuel Crespo [6,‡], Ciara K. O'Sullivan [4,8], Ezequiel Ruiz-Mateos [3] and Eva Poveda [1,*,‡]

1 Group of Virology and Pathogenesis; Galicia Sur Health Research Institute (IIS Galicia Sur). SERGAS-UVigo, 36213 Vigo, Spain; helena.codina@iisgaliciasur.es (H.C.); lucia.patino@iisgaliciasur.es (L.P.); maria.marcos@iisgaliciasur.es (M.M.B.); alexandre.perez@iisgaliciasur.es (A.P.)
2 Rare Diseases & Pediatric Medicine Research Group, Galicia Sur Health Research Institute (IIS Galicia Sur). SERGAS-Uvigo, 36213 Vigo, Spain; irene.vieitez@iisgaliciasur.es
3 Clinic Unit of Infectious Diseases, Microbiology and Preventive Medicine, Institute of Biomedicine of Seville, IBiS, Virgen del Rocío University Hospital/CSIC/University of Seville, 41013 Seville, Spain; Alicia.gutierrez.valencia@gamil.com (A.G.-V.); maria_tr_5@hotmail.com (M.T.-R.); anasernagallego@gmail.com (A.S.-G.); esperanzamunnozm@gmail.com (E.M.-M.); ezequiel.ruizmateos@gmail.com (E.R.-M.)
4 INTERFIBIO Consolidated Research Group, Departament d' Enginyeria Quimica, Universitat Rovira i Virgili, 43003 Tarragona, Spain; Vasso.Skouridou@urv.cat (V.S.); Mariluz.Botero@urv.cat (M.B.-G.); ciara.osullivan@urv.cat (C.K.O.)
5 Methodology and Statistics Unit, Galicia Sur Health Research Institute (IIS Galicia Sur)-Complexo Hospitalario Universitario de Vigo, SERGAS-UVigo, 36213 Vigo, Spain; cristina.martinez@iisgaliciasur.es
6 Infectious Diseases Unit, Department of Internal Medicine, Complexo Hospitalario Universitario de Vigo, IIS Galicia Sur, SERGAS-UVigo, 36213 Vigo, Spain; manuel.crespo.casal@sergas.es
7 Microbiology Service, Galicia Sur Health Research Institute (IIS Galicia Sur). SERGAS-Uvigo; 36213 Vigo, Spain; Jorge.julio.cabrera.alvargonzalez@sergas.es
8 Institució Catalana de Recerca i Estudis Avançats, 08010 Barcelona, Spain
* Correspondence: eva.poveda.lopez@sergas.es; Tel.: +34-986-21-74-63; Fax: +34-986-800-096
† These authors contributed equally to this work.
‡ On behalf of the Cohort COVID-19 of the Galicia Sur Health Research Institute.

Supplementary Methods

ddPCR Quantification

The one-step RT-PCR reaction mixture was assembled from 1x supermix, 20U/μL reverse transcriptase, 15mM DTT, 1x 2019-nCoV CDC ddPCR triplex probe assay, 5.5μL of RNA sample and RNase/DNase free water up to a final volume of 22μL. Then, the reaction mixture was converted to droplets with the QX200 droplet generator (Bio-Rad Laboratories). Droplet partitioned samples were amplified in a C1000 Touch™ Thermal Cycler (Bio-Rad Laboratories) under these cycling conditions: 50°C for 60 min (reverse transcription), 95°C for 10 min (enzyme activation), followed by 40 cycles of 94°C for 30 s (denaturation) and 55°C for 1 min (annealing and extension), 98°C for 10 min (enzyme deactivation), and 4°C for 30 min (droplet stabilization). The cycled plate was read in the FAM and HEX channels using the QX200 Reader (Bio-Rad Laboratories). All experiments included a positive SARS-CoV-2 control (Exact Diagnostics SARS-CoV-2 Standard, Exact Diagnostic), a negative control (Exact Diagnostics SARS-CoV-2 Negative, Exact Diagnostic) and a no template control. All controls and samples were tested in duplicate. The data analysis was performed using the QuantaSoft Analysis Pro Software (Bio-Rad Laboratories). As quality control, a result was accepted if it had equal or more than 10.000 droplets. In addition, a sample was considered positive for SARS-CoV-2, and a quantitative data was reported, if it had both a minimal concentration of N (N1 and/or N2) of 0.1 copies/μL of 1X ddPCR reaction, and two or more positive droplets for N1 and/or N2 were present.

For *RPP30*, the minimal concentration was up to 0.2 copies/μL of 1X ddPCR reaction and four or more positive droplets. Absolute quantification data was expressed in copies per milliliter of swab.

The serial dilutions of the RNA extracted from the commercial positive control for the experimental linearity study ranged from 200 copies/μL to 0.39 copies/μL for N1 and N2, and from 200 copies/μL to 0.78 copies/μL for *RPP30* (according to the minimal concentration established by the assay). The mean values obtained were compared with the expected values for each target (N1, N2 and *RP30*), and the results were expressed in $\log_{10}$ (copies/reaction). Finally, we also normalized the viral load of each clinical sample, referring the SARS-CoV-2 viral load quantification to the cellular quality of the swab (using the copy number of *RPP30* as reference for diploid cells), so the results were expressed in copies/10^4 cells for the viral load dynamic study. We also converted the SARS-CoV-2 viral load in $\log_{10}$ (copies/10^4 cells) in the graphics.

Supplementary Figures/Tables

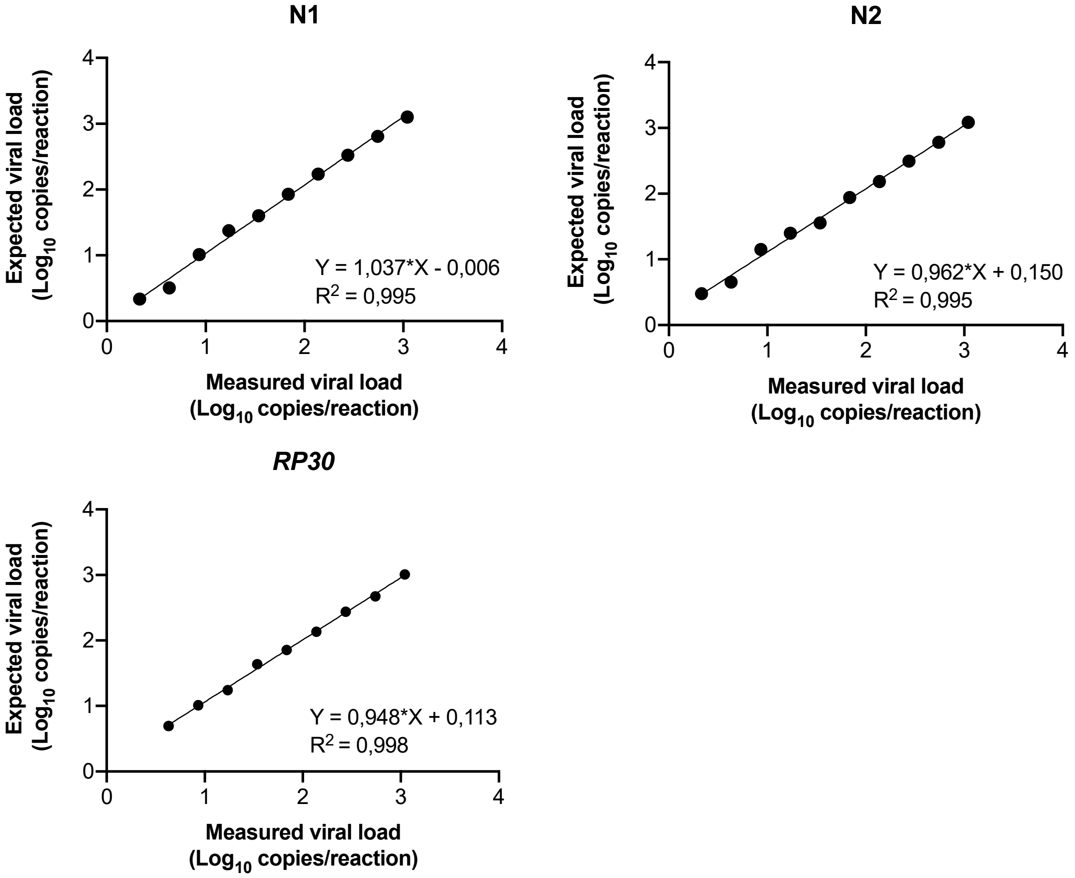

Figure S1. Linear regression analysis of the RT-ddPCR SARS-CoV-2 assay. The graphics show the linearity between the expected and the observed values quantified from the serial dilutions of the SARS-CoV-2 commercial control (Exact Diagnostics SARS-CoV-2 Standard, Exact Diagnostic) and for the three targets included in the assay (N1, N2 and RP30). The quantification is represented as copies of target per reaction (in Log10 format), and each black dot represents the mean of the replicates quantified for each dilution. Linear regression equation and correlation factor (r-square) are also included in the plots.

Table S1. IgA, IgM, IgG levels at the different points of the study period. Data are represented as median (IQR). n=18.

	IgA (µg/mL)	IgM (µg/mL)	IgG (µg/mL)
Baseline	6.72 (0.00–22.54)	0.56 (0.12–1.30)	26.67 (9.14–86.38)
Day 3	18.00 (7.43–32.28)	1.01 (0.54–2.43)	70.81 (38.96–133.50)
Day 6	26.25 (11.07–61.32)	2.21 (0.61–4.52)	109.30 (52.89–174.01)
Month 1	2.45 (0.27–6.97)	0.52 (0.08–0.88)	89.12 (61.40–114.05)
Month 3	0.00 (0.00–2.01)	0.10 (0.00–0.53)	63.70 (33.97–87.76)
Month 6	0.00 (0.00–0.49)	0.00 (0.00–0.15)	15.93 (9.88–25.01)

Table S2 Cytokine levels (severe vs. moderate). Data are median (IQR). Mann–Whitney U test was performed. P-values < 0,05 were considered significant and are highlighted in bold.

	Moderate Patients	Severe Patients	p-Value
	Baseline cytokines (pg/mL)		
IL-6	5.44 (2.93–11.63)	32.72 (20.78–107.92)	0.002
IL-8	6.43 (4.65–9.51)	19.99 (9.83–45.67)	0.007
IP-10	515.40 (141.30–633.13)	1083.80 (439.15–4613.25)	0.102
TNF-α	8.12 (7.31–11.56)	11.55 (10.40–17.45)	0.083
sCD25	1221.50 (775.33–2806.00)	2160.00 (1541.25–2877.00)	0.250
MIP-1β	15.37 (8.99–17,51)	25.29 (19.53–34.19)	0.001
IL-1β	1.50 (1.31–2.02)	1.65 (1.07–1.77)	0.750
MIP-1α	12.74 (7.95–15.47)	18.52 (5.02–24.21)	0.682
IFN-γ	35.91 (26.79–53.89)	47.59 (36.60–101.63)	0.213
	Month 1 cytokines (pg/mL)		
IL-6	2.40 (1.37–2.80)	1.08 (0.88–8.94)	0.635
IL-8	4.73 (3.47–7.06)	5.18 (3.04–6.13)	0.945
IP-10	67.57 (38.28–103.67)	107.30 (97.54–114.33)	0.106
TNF-α	7.75 (6.91–9.73)	6.98 (5.02–10.13)	0.635
sCD25	824.75 (742.48–997.3)	739.6 (201.06–1848.33)	0.839
MIP-1β	12.60 (11.30–15.71)	11.81 (7.08–25.45)	0.839
IL-1β	1.83 (1.22–2.74)	1.08 (0.90–1.44)	0.024
MIP-1α	17.88 (13.28–24.16)	14.48 (7.02–18.58)	0.347
IFN-γ	46.28 (41.57–57.21)	25.10 (21.74–33.28)	0.024
	Month 3 cytokines (pg/mL)		
IL-6	1.88 (0.84–2.81)	4.94 (4.45–5.42)	0.240
IL-8	5.21 (2.97–8.05)	2.03 (0.25–4.99)	0.060
IP-10	57.29 (24.32–131.25)	71.34 (47.64–99.31)	0.699
TNF-α	5.66 (4.72–6.70)	4.94 (4.45–5.42)	0.190
sCD25	730.40 (549.55–944.25)	782.5 (621.95–1092.50)	0.797
MIP-1β	12.24 (7.35–18.75)	5.27 (4.92–6.47)	0.012
IL-1β	1.75 (1.14–1.99)	0.26 (0.19–1.16)	0.029
MIP-1α	15.46 (11.23–23.14)	8.23 (0.15–10.70)	0.007
IFN-γ	50.80 (36.80–63.43)	28.93 (22.74–33.62)	0.029
	Month 6 cytokines (pg/mL)		
IL-6	2.44 (0.09–3.06)	1.21 (0.09–2.69)	0.660
IL-8	2.53 (1.14–4.09)	1.34 (0.69–7.74)	0.859
IP-10	42.97 (31.39–87.07)	62.86 (52.44–84.92)	0.256
TNF-α	6.15 (5.31–6.69)	5.61 (5.24–6.92)	0.733
sCD25	533.00 (486.80–714.40)	713.85 (535.00–989.75)	0.216
MIP-1β	9.52 (6.39–16.89)	8.54 (7.46–11.46)	1.000
IL-1β	1.86 (0.63–2.43)	0.78 (0.38–1.32)	0.145
MIP-1α	32.38 (24.26–44.49)	24.85 (8.46–25.39)	0.069
IFN-γ	60.12 (47.81–95.37)	48.17 (38.02–55.31)	0.098
